# The Impact of Teacher and Peer Support on Preservice EFL Teachers’ Work Engagement in Their Teaching Practicum: The Mediating Role of Teacher L2 Grit and Language Teaching Enjoyment

**DOI:** 10.3390/bs14090785

**Published:** 2024-09-06

**Authors:** Jiqun Fan, Xiaobing Lu, Qinqing Zhang

**Affiliations:** 1 School of Foreign Languages, Huainan Normal University, Huainan 232038, China; 106050@hnnu.edu.cn (J.F.); zhangqinqing@hnnu.edu.cn (Q.Z.); 2 Department of Applied Foreign Language Studies, Nanjing University, Nanjing 210023, China

**Keywords:** preservice teachers, teacher support, peer support, positive language emotions, work engagement

## Abstract

The academic study of work engagement among pre-service teachers during their practicum has been notably sparse, with even fewer investigations examining the influence of environmental stimuli on their engagement levels and the role of individual psychological and emotional factors throughout the practicum. To address this research gap, the present study, informed by the Stimulus–Organism–Response (SOR) theory, has developed a structural equation model. This model posits teacher support and peer support as stimuli, L2 grit and the foreign language teaching enjoyment as the organism, and the work engagement of pre-service teachers as the behavioral response. A quantitative survey was conducted among 516 preservice EFL teachers to examine the relationships among variables in the model. Findings showed direct predictions of work engagement by teacher support, L2 grit, and FLTE. L2 grit and FLTE sequentially mediated the relationship between teacher and peer support and work engagement. This study identified the factors influencing preservice EFL teachers’ work engagement, contributing to a deeper understanding of their psychological characteristics and emotional experiences during the teaching practicum. Additionally, the study offers practical implications for universities and internship schools to enhance preservice teachers’ work engagement during the practicum.

## 1. Introduction

Preservice teachers, commonly referred to as student teachers or future educators, constitute a distinct group within education due to their simultaneous roles as both learners and prospective teachers. The practicum, functioning as a crucial nexus between theory and practice, plays a pivotal role in the professional development of preservice teachers. Serving as a vital conduit between theoretical knowledge and practical application, the practicum is essential for preservice teachers to cultivate teaching expertise and skills [1]. Within the practicum, the subjective experiences of participants and the supportive community emerge as crucial determinants shaping preservice teachers’ development [2].

Regarding participant subjectivity, previous studies have emphasized the pivotal role of content and pedagogy in nurturing competent preservice teachers [3,4]. In recent years, an expanding body of research has shifted focus toward investigating various psychological aspects of preservice teachers, including their educational perceptions, professional commitment, cognitive competence, personal attitudes, and identity construction [5,6,7]. Among these psychological elements, the majority are closely related to the concept of work engagement. The latter generally refers to the relationship of the employee with his or her work, encompassing personal attributes such as vigor, dedication, and absorption that the employee exhibits in their work [8]. In the realm of educational research, work engagement emerges as a critical factor in the professional development and future career success of in-service teachers, influencing their job satisfaction, attitudes toward the teaching profession [9], and students’ academic engagement and social competence [10,11]. However, to date, prior research on work engagement among preservice EFL teachers during their practicum is limited. Therefore, empirical investigation into preservice teachers’ work engagement has theoretical significance for teacher education research and practical implications for societal progress.

On the other hand, according to the theory of Positive Psychology, positive emotions can foster upward mental states in individuals, thereby enhancing their engagement and subsequently improving their performance. However, research on the relationship between positive emotions and work engagement among pre-service teachers is relatively scarce. In light of the reality of pre-service EFL teachers, this study specifically selects teacher second language (L2) grit, which reflects the teachers’ perseverance and interest in EFL teaching, and the joyful sense experienced during EFL teaching, known as foreign language teaching enjoyment, to examine the impact of positive emotions on the work engagement of pre-service EFL teachers. Furthermore, similar to participant subjectivity, social support perceived by preservice teachers warrants considerable attention. Social support is an individual’s perception of receiving help and support from the outside world during social interactions. It can be categorized into four types based on the kind of support: emotional, appraisal, instrumental, and informational [12]. Additionally, social support can be differentiated based on its source into several types such as family support, school support, teacher support, and peer support [13]. Considering the practical conditions of pre-service teachers’ practicum, this paper primarily focuses on the impact of teacher support and peer support on the work engagement of pre-service teachers. As an established psychological theory, the Stimulus–Organism–Response (S-O-R) model posits that external stimuli influence an individual’s cognitive and affective components, which in turn affect the individual’s behavioral responses or tendencies [14].

Therefore, this study integrates empirical research and combines the S-O-R theory with Positive Psychology to investigate the impact of both teacher support and peer support on preservice teachers’ work engagement, mediated by L2 grit and foreign language teaching enjoyment (FLTE). The study underscores the importance of understanding the emotional factors influencing preservice teachers’ work engagement, particularly in the context of EFL teaching during the practicum. Moreover, it provides insights into the design of teaching practicum experiences and the preparation provided by universities and schools.

## 2. Literature Review

### 2.1. The Stimulus–Organism–Response Model

The S-O-R model posits that various environmental factors can serve as stimuli that affect an individual’s internal condition, known as the organism, thereby eliciting specific behavioral reactions [15]. Within education, this theory underscores the significance of external stimuli in shaping students’ cognitive and emotional responses, which subsequently influence their learning behaviors and satisfaction [16,17]. The S-O-R framework is particularly suitable for this study for two main reasons. First, it has been extensively utilized in educational contexts. For example, Zhang et al. integrated the Social Cognitive Career Theory with the S-O-R model to examine the relationships among social support systems, interaction relationships, self-efficacy, generic skills, and learning satisfaction [18]. Ge et al. proposed a model of the influence of teacher support on learning persistence in MOOCs by combining the S-O-R model with the Technology Acceptance Model [19]. Second, the variables under investigation in this study align with the typical components of the S-O-R framework. Both teacher and peer support can enhance an individual’s performance or mitigate negative impacts [20], thereby serving as significant predictors of student self-efficacy and academic performance [21]. The L2 grit and FLTE of preservice teachers in this study represent positive emotions that cultivate an optimistic and growth-oriented mindset among EFL learners [22]. Work engagement reflects pre-service teachers’ passion, concentration, and effort in teaching work. It indicates individual intentional tendencies or actual behaviors, essentially representing a coping response to external stimuli. Therefore, this study regards the pre-service EFL teachers’ perceived teacher support and peer support as stimuli, views the pre-service teachers’ L2 grit and foreign language teaching enjoyment as internal organismic states, and takes their work engagement as the response. By constructing a SOR predictive model for pre-service teachers’ work engagement, the study strives to explore the impact of teacher support and peer support on pre-service teachers’ work engagement, as well as the mediating roles of psychological emotions such as pre-service teachers’ L2 grit and foreign language teaching enjoyment.

### 2.2. Work Engagement

Work engagement encompasses involvement, passion, enthusiasm, absorption, focused effort, and energy. Initially conceptualized by Kahn, work engagement denotes the alignment of organization members’ selves with their work roles, fostering positive outcomes both for individuals and the organization [23]. Building on Kahn’s framework, Rothbard expanded the concept to include attention and absorption, defining it as a two-dimensional motivational construct. In contrast, other scholars view work engagement as the positive counterpart to burnout, representing a state of positive well-being in the workplace [24]. For instance, González-Romá and Bakke describe work engagement as a fulfilling state characterized by vigor, dedication, and absorption [25]. Within the realm of education, work engagement has been explored through two lenses: work-related resources and teachers’ personal attributes. Inspired by Positive Psychology, researchers have begun examining teachers’ positive emotions in relation to work engagement, including efficacy [26,27]. Despite this literature, research on preservice teachers’ work engagement remains scarce, calling for investigations into various aspects of their emotional experiences.

### 2.3. Teacher Support

Teacher support refers to the extent to which a teacher aids students in their learning endeavors [28]. Initially conceived as a single-dimensional construct encompassing emotional support for trust, interest, and encouragement [29], the concept has evolved into a multidimensional framework, including support for autonomy, structure, and involvement [30], or further categorized into emotional, appraisal, and instrumental supports [31]. Extensive research has highlighted the positive influence of teacher support on learners’ motivation, academic effort, classroom engagement, and academic achievement [32,33,34]. Moreover, studies indicate a strong correlation between EFL teacher support and students’ cooperative learning, positive emotions, learning experiences, motivation, and willingness to communicate [35,36,37,38]. In the context of the teaching practicum, teacher support refers to the assistance preservice teachers perceive from their mentor teachers, who play a pivotal role in their learning to teach [39]. Since mentor teachers are middle school teachers and are not affiliated with the university where the preservice teachers are enrolled, coupled with the fact that mentor teachers themselves have relatively heavy teaching workloads, the support provided by mentor teachers to preservice teachers differs from the traditional classroom teaching support and assistance provided by teachers to students. The assistance from mentor teachers is primarily manifested in emotional encouragement during activities such as classroom observations and teaching demonstrations. Although the mentor teacher is of great significance to pre-service teachers, there is currently a lack of research on the impact of teacher support on the work engagement of pre-service teachers.

### 2.4. Peer Support

Peer support entails the social and emotional backing individuals receive from their peers, based on respect, shared responsibility, and mutual agreement on what constitutes helpful assistance [40]. Widely explored in mental health services and considered in workforce policy-making [41], peer support holds significance in the educational realm, where it denotes the assistance and aid students provide one another in their learning endeavors.

Critical for student development, peer support plays an indispensable role in both mental and academic domains [42]. Facilitating the sharing of successes, fears, interests, and concerns among learners [43,44], it effectively alleviates learning anxiety [45]. Regarding its academic implications, peer support has been shown to enhance the quality of feedback and communication, thereby fostering successful collaborative learning opportunities [46,47]. Moreover, peer support has been found to bolster learners’ motivation, cultivate self-regulated learning skills, and mediate the relationship between teacher support and students’ self-efficacy [48,49,50].

In the EFL context, although limited in number, existing studies have revealed the positive impact of peer support in eliciting favorable language emotions [51], enhancing engagement in English learning [52], and alleviating language learning burnout [53].

### 2.5. Teacher L2 Grit

Grit, as a non-cognitive attribute, refers to one’s mental resilience in persistently tackling challenges and maintaining sustained effort and interest despite encountering setbacks [54]. It is conceptualized as a personality trait that extends beyond mere motivation [55] or conscientiousness [56]. Comprising two dimensions—perseverance of effort (PE) and consistency of interest (CI)—grit has been expanded to teaching contexts by Sudina et al. (2021), who described language teacher L2 grit as a personality trait determining educators’ perseverance and sustained interest in achieving instructional objectives [57]. Similar to students’ L2 grit in foreign language learning, the grit of EFL teachers also holds paramount importance, as successful foreign language teaching requires teachers’ persistence, enthusiasm, and vitality [58].

Existing literature highlights the myriad benefits of teachers’ L2 grit on both educators and students, as well as learning activities. For instance, Maiers and Sandvold discovered that gritty teachers exhibit stronger passion and commitment to their work [58]. Duckworth et al. suggested that EFL teachers’ grit enhances their job satisfaction and fulfillment [59]. Furthermore, previous research has explored a positive association between teachers’ L2 grit and various traits among EFL educators, including a language growth mindset, willingness to communicate, and creativity [60,61]. However, due to the novelty of this construct, further investigation into language teachers’ L2 grit and its correlations, particularly its relationship with FLTE among language educators, are warranted [62].

### 2.6. Foreign Language Teaching Enjoyment

Enjoyment, a fundamental construct in Positive Psychology (PP), encompasses happiness, interest, pride, fun, and a sense of meaning [63]. Within the EFL context, this notion is elaborated as foreign language enjoyment (FLE), representing a complex emotion that reflects the human drive for success amid challenging tasks [57]. Dewaele and MacIntyre empirically identified 21 items on the FLE scale, categorized into two components: social FLE and private enjoyment [64].

In terms of its impact on EFL learning, prior research indicates that FLE significantly contributes to foreign language education by enhancing learners’ motivation, willingness to communicate, language proficiency, and ultimately, academic achievements [65,66,67]. To describe teachers’ enjoyment in FL teaching, Ergün and Dewaele introduced foreign language teaching enjoyment (FLTE), reflecting a teacher’s ability to foster a supportive learning environment and cultivate satisfactory teacher–student interaction [68]. Despite its novelty, FLTE has garnered scholarly attention, with studies revealing that differences in gender and place of residence do not predict FLTE [69]. Moreover, teacher resilience emerges as a significant predictor of FLTE [68], and FLTE has been shown to alleviate teachers’ burnout and fatigue and enhance their work engagement [70,71]. However, research on preservice teachers’ FLTE remains largely unexplored.

### 2.7. The Present Study

This study examines the interrelationship among teachers and peer support, teacher L2 grit (L2G), foreign language teaching enjoyment (FLTE), and work engagement. These variables are analyzed within the framework of the S-O-R paradigm from the perspective of Positive Psychology. Mentor teachers’ supportiveness during preservice teachers’ teaching practicum has been noted to accelerate positive changes [72], while preservice teachers also foster each other’s growth through activities such as peer coaching, assistance, feedback, and assessment [73,74]. Consequently, this study integrates Positive Psychology with the S-O-R model to examine preservice teachers’ work engagement as a psychological response and explore the potential influencing factors.

**Figure 1:**
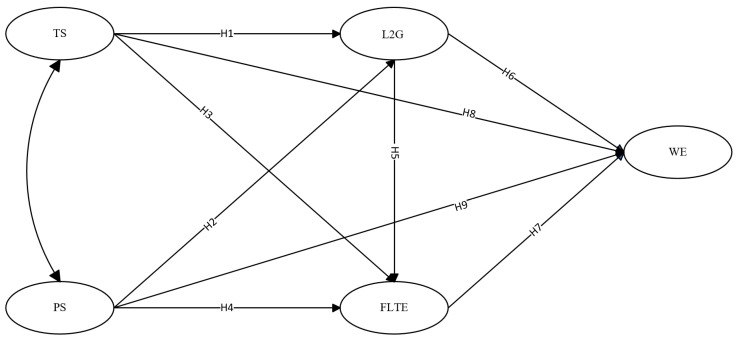
Hypothesized model.

Building upon existing literature and the aforementioned theoretical framework, the hypotheses of the model (see Figure 1) are formulated as follows:

**H1.**  *Teacher support positively influences preservice teachers’ L2 grit.*

**H2.**  *Peer support positively influences preservice teachers’ L2 grit.*

**H3.**  *Teacher support positively influences preservice teachers’ foreign language teaching enjoyment*.

**H4.**  *Peer support positively influences their foreign language teaching enjoyment.*

**H5.**  *Preservice teachers’ L2 grit positively influences their foreign language teaching enjoyment*.

**H6.**  *Preservice teachers’ L2 grit positively influences their work engagement*.

**H7.**  *Preservice teachers’ foreign language teaching enjoyment positively influences their work engagement*.

**H8.**  *Teacher support exerts a positive and direct influence on preservice teachers’ work engagement*.

**H9.**  *Peer support exerts a positive and direct influence on preservice teachers’ work engagement*.

**H10.**  *Preservice teachers’ L2 grit acts as a mediator between teacher support and their work engagement*.

**H11.**  *Preservice teachers’ L2 grit mediates peer support and their work engagement*.

**H12.**  *Preservice teachers’ foreign language teaching enjoyment acts as a mediator between teacher support and their work engagement*.

**H13.**  *Preservice teachers’ foreign language teaching enjoyment acts as a mediator between peer support and work engagement*.

**H14.**  *Preservice teachers’ L2 grit and foreign language teaching enjoyment mediate the relationship between teacher support and their work engagement*.

**H15.**  *Preservice teachers’ L2 grit and foreign language teaching enjoyment mediate the relationship between peer support and their work engagement*.

## 3. Method

### 3.1. Participants and Procedure

The study sample comprised 516 fourth-year students majoring in English from five universities in Anhui Province, China. These universities all offer teacher education programs, cultivating English teachers for primary and secondary schools in the area. All participants had just completed a minimum of six months of teaching practicum in junior middle schools. The sample consisted of 341 females and 175 males. Among the participants, more than half were 22 years old (51.9%), followed by 21-year-olds at 23.8%, 23-year-olds at 22.7%, and those aged 24 years and above making up 1.6% of the sample.

As the respondents were from different areas of Anhui Province, the researchers chose to conduct the survey electronically. Participants were recruited through convenience sampling, with ethics approval and informed consent obtained initially. To preserve the originality of the constructs, the scales were not translated into Chinese. Questionnaires were distributed via WeChat, with a QR code sent to 600 Chinese EFL preservice teachers in February 2024. After two weeks, 516 valid forms were collected, resulting in an 86% return rate, with no missing data.

### 3.2. Instruments

The constructs in this study include teacher support (TS), peer support (PS), teacher second language grit (L2 grit), foreign language teaching enjoyment (FLTE), and teachers’ work engagement (WE). All the items were rated on a 5-point Likert scale ranging from 1 (“strongly disagree”) to 5 (“strongly agree”).

According to the current status of the teaching practicum, guidance from secondary school mentor teachers was not a classroom lecture but teaching observation and demonstrating teaching, helping interns establish teaching plans and refine lesson plans, thus providing guidance on preservice teachers’ performance during the practicum. Considering this, the study employed the teacher support subscale developed by the Assessment Research Center of a Hong Kong Educational Institution [75], consisting of four items (e.g., “Mentor teachers are willing to help me”). The items reflect the support and assistance secondary school teachers provided to preservice teachers in terms of encouragement, teaching guidance, and comfort in actual teaching practice. The Cronbach’s α coefficient for the scale was 0.927, indicating high internal consistency. Additionally, fit indexes (χ^2^/df = 1.170, GFI = 0.998, AGFI = 0.989, CFI = 1.000, TLI = 0.999, RMSEA = 0.018, SRMR = 0.0051) supported the validity of the scale.

During the practicum, due to the limited capacity of each internship school, English major student teachers were often assigned to a particular school alongside students from other majors, and they were grouped by class. They may not have known each other. During the practicum, they observed each other‘s classes and offered encouragement and support. Therefore, peer support was assessed using a modified scale from Zheng and Zhou, comprising four items (e.g., “My teammates like me the way I am”) [76]. The Cronbach’s α coefficient for the scale was 0.922, indicating strong internal consistency. The fit index of the scale was χ^2^/df = 2.681, GFI = 0.995, AGFI = 0.975, CFI = 0.998, TLI = 0.993, RMSEA = 0.057, SRMR = 0.0081, supporting the validity of the scale.

The present study utilized the L2 grit scale developed by Teimouri et al. to assess participants’ levels of L2 grit [77]. This scale consists of nine items categorized into two dimensions: perseverance of effort (PE, four items, e.g., “I put much time and effort into overcoming my foreign language weaknesses”) and consistency of interest (CI, five items, e.g., “I am not interested in teaching a foreign language as I used to be”). In this study, Cronbach’s α values for PE and CI were 0.856 and 0.855, respectively, while the overall reliability of the scale was 0.876. The fit indexes (χ^2^/df = 2.681, GFI = 0.995, AGFI = 0.975, CFI = 0.998, TLI = 0.993, RMSEA = 0.057, SRMR = 0.0081) indicate good validity of the scale.

FLTE was measured using a scale adapted from Ergün and Dewaele [68], comprising nine items (e.g., “In class, I feel proud of my accomplishments”). In the survey, participants were reminded that the classroom scenarios referred to in the variable items pertain to the classes they were teaching during their practicum. In this study, the Cronbach’s α was 0.945, indicating high internal consistency. Furthermore, the fit indexes of this scale (χ^2^/df = 2.202, GFI = 0.975, AGFI = 0.958, CFI = 0.991, TLI = 0.988, RMSEA = 0.048, SRMR = 0.0174) suggest good validity.

Work engagement was evaluated using the long version of the Utrecht Work Engagement Scale (UWES-N) [78], which comprises three dimensions: vigor (VI, 6 items, e.g., “At my work, I feel bursting with energy”), dedication (DE, 5 items, e.g., “I am enthusiastic about my job”), and absorption (AB, 6 items, e.g., “When I am working, I forget everything else around me”). The Cronbach’s α values for these dimensions were 0.910 (AB), 0.892 (DE), and 0.891 (VI), with an overall Cronbach’s α of 0.934. Furthermore, the fit indexes (χ^2^/df = 1.753, GFI = 0.956, AGFI = 0.942, CFI = 0.983, TLI = 0.981, RMSEA = 0.038, SRMR = 0.0278) suggest good validity of the measurement.

In the present study, all items exhibited factor loadings well above the threshold level of 0.50 [79]. Additionally, the obtained alpha and CR values for all constructs surpassed 0.70, indicating high internal consistency reliability [79].

The average variance extracted (AVE) for all constructs ranged from 0.576 to 0.763, surpassing the threshold value of 0.50, thus confirming acceptable convergent validity (see Table A1). Furthermore, the estimated interrelations among all constructs were less than the square roots of the AVE for each construct (see Table A2), suggesting that discriminant validity was not an issue in this study [80].

### 3.3. Data Analysis

Data analysis employed SPSS 26.0 and AMOS 24.0. Descriptive and correlation analyses were initially conducted to present an overview of the data. Confirmatory factor analysis was then used to confirm the measurement model, followed by structural equation modeling (SEM) to assess the fit of the hypothesized model and to report regression and mediation. The goodness-of-fit indexes adhered to criteria such as χ^2^/df ≤ 3, CFI, GFI, AGFI, and TLI ≥ 0.90, RMSEA ≤ 0.08, and SRMR ≤ 0.10 [81].

## 4. Results

### 4.1. Descriptive and Correlation Analysis

As shown in Table 1, preservice teachers reported moderate levels of teacher support, peer support, L2 grit, FLTE, and work engagement, with scores of 3.393, 3.550, 3.176, 3.297, and 3.357 out of 5, respectively. Additionally, all latent variables displayed acceptable levels of skewness and kurtosis, and demonstrated significant positive correlations (r > 0.300, *p* < 0.01) among themselves (see Table 1).

### 4.2. Hypothetical Model

**Figure 2:**
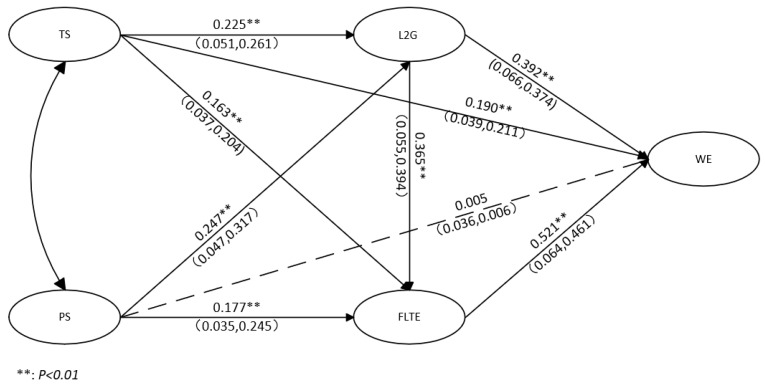
Path analysis.

This study constructed a structural model with teacher support and peer support as antecedent variables, teacher L2 grit and FLTE as mediating variables, and teachers’ work engagement as the outcome variable (as shown in Figure 2). The test of the structural model indicated that the model fit indexes were satisfactory (χ^2^ = 1068.818, df = 845, χ^2^/df = 1.265, GFI = 0.913, AGFI = 0.902, CFI = 0.985, TLI = 0.984, IFI = 0.986, RMSEA = 0.023, SRMR = 0.033).

As shown in Figure 2, both teacher support (TS) and peer support (PS) positively predicted L2 grit (β = 0.261, *p* < 0.01, SE = 0.051; β = 0.317, *p* < 0.01, SE = 0.047), thereby supporting H1 and H2, respectively. Additionally, both TS and PS positively affected foreign language teaching enjoyment (FLTE) (β = 0.204, *p* < 0.01, SE = 0.037; β = 0.245, *p* < 0.01, SE = 0.035), supporting H3 and H4, respectively. Furthermore, L2 grit positively predicted FLTE (β = 0.394, *p* < 0.01, SE = 0.055), supporting H5. On the other hand, both L2 grit and FLTE positively predicted work engagement (L2G-WE: β = 0.374, *p* < 0.01, SE = 0.066; FLTE-WE: β = 0.461, *p* < 0.01, SE = 0.064), supporting H6 and H7. Finally, TS positively affected work engagement (β = 0.211, *p* < 0.01, SE = 0.039), supporting H8. However, the direct effect of PS on work engagement was not significant (β = 0.006, *p* = 0.89, SE = 0.036), leading to the rejection of H9.

Guided by Preacher and Hayes, the present study conducted bootstrapping to test for indirect effects and mediation [82]. First, as shown in Table 2, the findings demonstrated that both teacher and peer support had significant indirect effects on work engagement (WE) through L2 grit, thus supporting H10 and H11. Second, teacher and peer support also had significant indirect impacts on WE via FLTE, supporting both H12 and H13. Third, both teacher and peer support respectively exerted significant indirect effects on work engagement via L2 grit and FLTE as chain mediators. These findings indicated support for H14 and H15.

## 5. Discussion

This study delves into the interplay model among teacher support, peer support, teacher L2 grit, foreign language teaching enjoyment, and preservice teachers’ work engagement. Existing literature has highlighted a scarcity of studies on the work engagement of preservice teachers, making the findings from this study worthy of careful consideration.

Firstly, the study demonstrates the positive impact of both teacher and peer support as stimuli on the positive emotions of preservice teachers. It aligns with the S-O-R paradigm which posits that feelings or emotions, as common organismic reactions, are the natural outcomes of exposure to environmental stimuli [83]. The current study shows that teacher and peer support can predict 25% of the changes in preservice teachers’ L2 grit. These results echo previous studies suggesting that teacher support can significantly bolster students’ resilience and persistence and foster academic interest [84,85]. Similarly, peer support can effectively enhance an individual’s persistence [86]. According to the proposed model in this study, teacher support, peer support, and L2 grit together can predict 45.9% of the changes in preservice teachers’ FLTE. This finding is consistent with previous studies indicating that teacher support could contribute to students’ enjoyment of foreign languages [87,88].

Secondly, the current study affirms the positive influence of preservice teachers’ emotions on their work engagement. It aligns with Positive Psychology, which posits that positive emotions toward foreign languages enhance language achievement and linguistic progress [89]. The findings echo those of Liu et al. and Noughabi et al., who established a significant link between EFL teachers’ L2 grit and their engagement [90,91]. While the role of foreign language enjoyment in teaching is less explored, the study’s findings are supported by Zhang et al., who demonstrated that heightened levels of FLTE are conducive to increased grit and engagement among preservice teachers [92]. This underscores the reciprocal nature of emotional factors and work engagement within the educational context.

Thirdly, this study elucidates the mediating function of L2 grit and FLTE, affirming their role as organismic factors in the pathway from teacher and peer support to preservice teachers’ work engagement. In previous studies employing the S-O-R model, emotion and experience have been identified as mediators in the relationship between stimulus and response [93,94]. The findings corroborate with those of Li and Sadoughi, and Hejazi, who recognized L2 grit as a mediator in the influence of teacher support on student engagement [95,96]. Similarly, Liu et al. and Dewaele and Li highlighted the mediation of FLTE in the impact of teacher support on student engagement, reinforcing the significance of L2 grit and FLTE in the motivational process of preservice teachers [63,97]. The present study also reveals the impact of L2 grit on FLTE in the proposed model and indicates the sequential mediation effect of L2 grit and FLTE in predicting preservice teachers’ work engagement.

Finally, concerning direct effects, the study reveals that teacher support significantly influences preservice teachers’ work engagement. This finding aligns with Lipscomb et al., who argue that workplace resources such as professional support can positively predict teachers’ work engagement [98]. However, the research also indicates that peer support does not significantly affect preservice teachers’ work engagement. This could be attributed to the current teaching practicum setup, where preservice teachers are spread across various primary and secondary schools, limiting peer communication and support in fostering their work engagement.

## 6. Conclusions and Implications

Through a survey of 516 preservice teachers during their educational practicum, the established model accounted for 76.7% of the variance in preservice teachers’ work engagement. As a result, the model is deemed instrumental in enhancing our understanding of the impact of external stimuli on work engagement, mediated by the positive mindset of preservice teachers.

The study not only confirmed the proposed model, but also successfully applied the S-O-R model to the field of teacher education. It innovatively integrated Positive Psychology to examine the influence of L2 grit and foreign language teaching enjoyment on preservice teachers’ work engagement, thereby enriching the theoretical discourse and piquing scholarly interest in the emotional dimensions of foreign language teaching within the context of preservice education. Additionally, the research delineated the mediating role of preservice teachers’ L2 grit and foreign language teaching enjoyment between the social support they perceive and their engagement as student teachers, offering a significant theoretical contribution to the extant literature.

The practical implications of this study are manifold. Firstly, it established a direct, positive correlation between teacher support and preservice teachers’ work engagement. Educational entities, such as universities and schools hosting practicum, are encouraged to bolster support for preservice teachers. Universities might consider crafting comprehensive programs to foster mentor teachers’ engagement in students’ practicum, while schools should emphasize the mentors’ pivotal role in providing timely guidance to preservice teachers facing challenges during teaching practice. Secondly, the study underscores the significance of positive emotions in fostering preservice teachers’ engagement in educational practice. Institutions and educators are advised to enhance psychological counseling aimed at cultivating a positive mindset among preservice teachers, encompassing traits such as perseverance, enthusiasm, and drive. Lastly, while the study found the direct impact of peer support on work engagement to be marginal, it suggests a need to refine the current practicum framework to bolster peer support mechanisms. Initiatives such as regular internal meetings could foster closer and more frequent interactions among preservice teachers, enabling them to seek and provide comfort, inspiration, and guidance, and consequently enhance their motivation and performance during the practicum [99].

Despite its contributions, the study acknowledges its limitations, presenting opportunities for future research. Teacher support is essential for the development of preservice teachers. The current use of a simplified scale may not fully capture the complexity of this issue. To better explore the potential impacts of multi-dimensional teacher support, future research will consider employing a long, multi-dimensional scale to focus on the intrinsic relationship between teacher support and the development of pre-service teachers. Furthermore, subsequent research could incorporate qualitative methods such as interviews and focus group discussions to effectively explore the role of psychological and emotional factors among preservice EFL teachers during their practicum. Finally, considering that family support is also a significant social stimulus for teachers [100], future research could incorporate family support to provide a more comprehensive understanding.

## Figures and Tables

**Table 1:** Descriptive statistics and correlation.

	1	2	3	4	5
TS (1)	1				
PS (2)	0.455 **	1			
L2G (3)	0.341 **	0.365 **	1		
FLTE (4)	0.463 **	0.490 **	0.482 **	1	
WE (5)	0.520 **	0.446 **	0.547 **	0.686 **	1
Mean	3.393	3.550	3.176	3.297	3.357
SD	0.976	1.115	1.007	0.794	0.914
Skewness	−0.783	−0.627	−0.371	−0.789	−0.534
Kurtosis	−0.004	−0.739	−1.006	0.162	−0.175

Note: ** *p* < 0.01; SD = standard deviation; TS: teacher support, PS: peer support, L2G: L2 grit, FLTE: foreign language teaching enjoyment, WE: work engagement.

**Table 2:** Mediation analysis.

Effect Type	Path Relationship	Point Estimate	Product of Coefficient		Bootstrapping (Bias-Corrected 95% CI)		
			SE	Z	Lower	Upper	*p*
Mediation Effect Tests							
DistalIE	TS-L2G-FLTE-WE (H14)	0.043	0.013	3.308	0.020	0.073	0.000
L2GIE	TS-L2G-WE (H10)	0.088	0.026	3.385	0.044	0.149	0.000
FLTEIE	TS-FLTE-WE (H12)	0.085	0.027	3.148	0.038	0.143	0.001
DE	TS-WE (H8)	0.190	0.041	4.634	0.112	0.274	0.000
DistalIE	PS-L2G-FLTE-WE (H15)	0.047	0.013	3.615	0.026	0.078	0.000
L2GIE	PS-L2G-WE (H11)	0.097	0.027	3.593	0.053	0.159	0.000
FLTEIE	PS-FLTE-WE (H13)	0.092	0.024	3.833	0.051	0.143	0.000
DE	PS-WE (H9)	0.005	0.039	0.128	−0.070	0.082	0.902

Note: DistalIE: distance indirect effect; L2GIE: indirect effect through L2G; FLTEIE: indirect effect through FLTE; DE: direct effect; TS: teacher support, PS: peer support, L2G: L2 grit, FLTE: foreign language teaching enjoyment, WE: work engagement.

## Data Availability

Data are available on request due to restrictions (e.g., privacy, legal, or ethical reasons).

## Appendix A

**Table A1:** Factor Analysis, Convergent Reliability, and Convergent Validity of the Measurement Model.

Construct	Items	Estimate	S.E.	Z-Value	P	Factor Loading	α	CR	AVE
PS	PS1	1				0.880	0.922	0.923	0.749
	PS2	1.014	0.038	26.938	***	0.870			
	PS3	1.067	0.039	27.338	***	0.877			
	PS4	0.937	0.038	24.974	***	0.835			
TS	TS1	1				0.890	0.927	0.928	0.763
	TS2	0.989	0.036	27.8	***	0.871			
	TS3	0.981	0.034	28.975	***	0.889			
	TS4	0.925	0.036	25.986	***	0.842			
PE	PE1	1				0.835	0.876	0.876	0.639
	PE2	0.866	0.044	19.53	***	0.777			
	PE3	0.93	0.048	19.487	***	0.776			
	PE4	0.927	0.045	20.509	***	0.807			
CI	CI1	1				0.775	0.885	0.885	0.607
	CI2	1.064	0.057	18.81	***	0.801			
	CI3	0.967	0.055	17.609	***	0.757			
	CI4	1.008	0.057	17.746	***	0.762			
	CI5	1.037	0.055	18.76	***	0.800			
DE	DE1	1				0.760	0.892	0.892	0.623
	DE2	1.104	0.059	18.81	***	0.808			
	DE3	1.074	0.058	18.419	***	0.793			
	DE4	1.083	0.058	18.752	***	0.806			
	DE5	1.027	0.057	18.02	***	0.778			
VI	VI1	1				0.770	0.891	0.89	0.576
	VI2	0.953	0.056	16.976	***	0.731			
	VI3	0.983	0.053	18.391	***	0.783			
	VI4	0.912	0.054	17.034	***	0.733			
	VI5	0.996	0.053	18.689	***	0.794			
	VI6	0.901	0.052	17.193	***	0.739			
AB	AB1	1				0.821	0.91	0.91	0.627
	AB2	0.967	0.047	20.721	***	0.797			
	AB3	0.94	0.046	20.357	***	0.787			
	AB4	0.956	0.047	20.157	***	0.782			
	AB5	0.902	0.046	19.503	***	0.763			
	AB6	0.931	0.045	20.739	***	0.798			
FLTE	FLTE1	1.013	0.045	22.399	***	0.824	0.945	0.945	0.658
	FLTE2	0.961	0.046	21.122	***	0.792			
	FLTE3	1				0.819			
	FLTE4	0.999	0.047	21.246	***	0.795			
	FLTE5	0.958	0.047	20.425	***	0.774			
	FLTE6	0.978	0.047	20.784	***	0.783			
	FLTE7	1.068	0.046	23.062	***	0.840			
	FLTE8	1.054	0.046	22.969	***	0.835			
	FLTE9	0.996	0.044	22.855	***	0.838			

Note: TS: teacher support, PS: peer support, L2G: L2 grit, FLTE: foreign language teaching enjoyment, WE: work engagement. ***: *p* < 0.001.

**Table A2:** Correlation Matrices and Discriminant Validity.

Construct	AB	FLTE	CI	VI	DE	PE	TS	PS
AB	0.792							
FLTE	0.623	0.811						
CI	0.425	0.485	0.779					
VI	0.648	0.627	0.420	0.759				
DE	0.606	0.645	0.559	0.674	0.789			
PE	0.426	0.443	0.625	0.396	0.548	0.799		
TS	0.493	0.488	0.360	0.479	0.465	0.295	0.873	
PS	0.428	0.520	0.350	0.377	0.434	0.355	0.489	0.865

Note: Square roots of the AVE are presented as diagonal elements.
